# Neuroanatomical signatures of chronotype in young adults

**DOI:** 10.1007/s11682-026-01153-7

**Published:** 2026-04-25

**Authors:** Iman Beheshti, Odelia Elkana

**Affiliations:** 1 Department Electrical Engineering Technology, Red River College Polytechnic, W406-160 Princess St, R3B 1K9 Winnipeg, MB Canada; 2 Behavioral Sciences, The Academic College of Tel Aviv-Yaffo, P.O.B 8401, Tel Aviv, 61083 Israel

**Keywords:** Chronotype, Circadian rhythm, Brain structure, Cortical thickness, Gray matter, Sleep health, Young adults, Neuroimaging

## Abstract

Despite extensive evidence linking chronotype to behavioral and physiological outcomes, its structural neuroanatomical correlates, especially in healthy young adults, remain insufficiently characterized, and multimodal structural investigations integrating Voxel-based morphometry (VBM), cortical thickness (CT), and brain-age metrics are still limited. We examined whether chronotype—preference for early or late sleep–wake timing—is associated with structural brain variation in 136 healthy young adults (68 early chronotypes [EC], 68 late chronotypes [LC]) using high-resolution MRI. Early and late chronotypes were defined using the Morningness–Eveningness subscale of the Chronotype Questionnaire (ChQ-ME): early chronotype (EC) scores 11–21 and late chronotype (LC) scores 22–32. The VBM analyses were conducted to assess gray and white matter morphology, complemented by CT analyses and estimation of brain-predicted age difference (Brain-PAD) as an index of biological brain aging. Sensitivity analyses additionally modeled ChQ-ME as a continuous predictor. Primary voxel-wise VBM analyses did not identify between-group differences in gray or white matter morphology that survived family-wise error (FWE) correction (*p* < 0.05). In pre-specified exploratory analyses (voxel-wise *p* < 0.001, uncorrected; cluster-level false discovery rate [FDR] correction q < 0.05), late chronotype (LC) participants showed an exploratory left cerebellar/occipital cluster with lower gray matter volume. Region-wise CT differences were nominal (*p* < 0.05) and did not survive FDR correction across regions. No significant differences in Brain-PAD were observed. In healthy young adults, chronotype-related structural differences were not detectable under conservative voxel-wise FWE correction; however, pre-specified exploratory analyses suggested a regionally specific cerebellar gray matter pattern and nominal CT trends. These findings motivate larger and longitudinal studies with objective sleep–wake timing measures to clarify whether sleep timing is linked to early structural variation and to test its potential modifiability.

## Introduction

Circadian rhythms govern virtually every aspect of human physiology, including sleep–wake timing, hormonal secretion, metabolism, and cognitive and emotional function (Jones et al. 2019). Chronotype, the individual preference for early (“morning type”) or late (“evening type”) activity, reflects endogenous circadian biology (Zou et al. 2022). Early chronotypes (EC) typically wake and perform optimally in the morning, whereas late chronotypes (LC) peak later in the day. These patterns are influenced by genetic factors, including polymorphisms in clock genes such as *PER3*, and show developmental shifts—toward eveningness in adolescence and morningness with aging—along with modest sex differences favoring earlier schedules in females (Zheng et al. 2024). Modern lifestyles frequently impose misalignment between biological rhythms and environmental demands. Artificial lighting, technology use, and rigid work or academic schedules create “social jetlag,” particularly affecting late chronotypes (McMahon et al. 2019, Zhang et al. 2019). This misalignment has been linked to sleep debt, emotional dysregulation, cognitive impairments, and altered neuroendocrine and immune function, including disrupted cortisol and melatonin rhythms and elevated inflammatory markers (Montaruli 2021, Gorfine et al. 2006). Chronic circadian disruption is also associated with elevated risk for psychiatric, metabolic, and neurodegenerative disorders (McMahon et al. 2019, Anothaisintawee et al. 2017, Taillard et al. 2021). Functional brain imaging has shown that sleep timing preferences (chronotype) are not merely behavioral traits but are closely linked to dynamic changes in brain function, including cortical excitability, synaptic plasticity, and cognitive processing (Salehinejad et al. 2021).

Despite growing recognition of chronotype’s impact on behavior and physiology, its structural neural correlates remain incompletely characterized—especially in healthy young adults, a population uniquely vulnerable to early circadian misalignment with potential long-term consequences. Few studies have examined how chronotype affects brain morphology, and comprehensive analyses integrating multiple structural measures are lacking. Emerging neuroimaging evidence indicates chronotype-related differences in gray matter (GM) volume in regions such as the precuneus, insula, thalamus, orbitofrontal cortex, and lateral occipital cortex (Horne and Norbury 2018, Horne and Norbury 2018, Evans et al. 2021). In contrast, white matter (WM)—essential for network integration and circadian regulation—has been largely overlooked. Specific brain regions are of particular interest. The anterior cingulate cortex (ACC) mediates emotional regulation and cognitive control, functions often disrupted in LC individuals. The occipital cortex, beyond visual processing, contributes to circadian light sensitivity and melatonin suppression, potentially showing chronotype-dependent structural variation (Grill-Spector et al. 2001, Malach et al. 1995, Lucas et al. 2014). The cerebellum, traditionally linked to motor coordination, is increasingly recognized for higher-order cognitive and affective roles but has received little attention in chronotype research (Schmahmann 2019, Stoodley and Schmahmann 2009). Because chronotype-related structural differences in healthy young adults may be subtle and spatially distributed, a multimodal structural approach may be necessary to improve sensitivity and interpretability. Accordingly, in addition to region-specific morphometric analyses, we incorporated brain age estimation (Brain-PAD) as a whole-brain summary index of neurobiological aging and vulnerability (Mishra et al. 2021, Sone and Beheshti 2022, Ball et al. 2021, Tesli et al. 2022). As chronotype is inherently continuous, we complemented the extreme-group comparison with sensitivity analyses modeling ChQ-ME as a continuous predictor across imaging outcomes.

To address these gaps, we conducted a comprehensive multimodal analysis of brain structure in healthy young adults with EC and LC. High-resolution MRI data were used to assess complementary aspects of brain morphology: voxel-based morphometry (VBM) to quantify GM and WM volumes in a whole-brain, unbiased framework; cortical thickness (CT) analysis to capture cortical integrity that may not be reflected by volume alone; and brain-age/Brain-PAD estimation to test whether chronotype relates to a whole-brain index of neurobiological variation. Finally, we examined associations between chronotype-related measures and sleep indices—sleep quality and daytime sleepiness—in relation to structural brain measures.

Based on prior literature, we tested whether LC individuals show subtle, regionally specific differences in GM volume and CT—particularly in the ACC, occipital cortex, and posterior cerebellar lobules—and explored whether chronotype relates to Brain-PAD and to sleep-related measures. By integrating multiple structural MRI modalities within the same young adult cohort and complementing categorical group comparisons with continuous chronotype sensitivity analyses, this study aims to clarify whether circadian timing is associated with subtle neuroanatomical variation in young adulthood and to inform hypotheses for future longitudinal research.

## Materials and methods

### Participants and MRI acquisition

We conducted a cross-sectional MRI study to examine structural brain differences related to chronotype in healthy young adults. Data were obtained from the OpenNeuro dataset (https://openneuro.org/datasets/ds003826/versions/3.0.1/file-display/dataset_description.json accessed March 1, 2025), including 68 EC and 68 age- and sex-matched LC. Participants were 19–35 years old, right-handed, had normal or corrected vision, and no neurological or psychiatric history. None were on medication. To minimize circadian disruption, individuals with shift work in the past six months or recent travel across more than two time zones were excluded. Daytime alertness and sleep quality were assessed via the Epworth Sleepiness Scale (ESS ≤ 10) and Pittsburgh Sleep Quality Index (PSQI ≤ 5), used as inclusion thresholds to ensure normal wakefulness and good subjective sleep. Participants reported regular sleep patterns (6–9 h/night). Chronotype was determined using the Chronotype Questionnaire (ChQ) (Oginska et al. 2017, Ogińska 2011). The morningness-eveningness subscale (ChQ-ME) assessed time-of-day preference, while the amplitude subscale (ChQ-AM) reflected circadian rhythm strength. Participants were classified as early chronotype ) EC; ChQ-ME 11–21) or late chronotype ) LC; ChQ-ME 22–32), using previously established cutoffs (Zareba et al. 2022). We adopted an extreme-group enrichment approach rather than a near-median split: participants were selected to represent clearly separated early vs. late diurnal preference profiles and were tightly matched on age and sex. To avoid reliance on categorization alone, we additionally modeled ChQ-ME as a continuous predictor in regression analyses for all primary imaging outcomes (sensitivity analyses).All self-report measures were completed before MRI acquisition.

Anatomical scans were collected on a 3T Siemens Skyra scanner using a GR_IR sequence (TR = 2.3 s, TE = 2.98 ms, TI = 0.9 s, flip angle = 9°, slice thickness = 1.1 mm, matrix = 248 × 256, FOV = 24 × 24 cm, 180 slices, scan time = 3.19 min). Additional details are provided in (Zareba et al. 2022). Preprocessing was conducted using CAT12 (http://www.neuro.uni-jena.de/cat/, accessed February 20, 2025), an SPM25 extension (https://www.fil.ion.ucl.ac.uk/spm/software/spm25/, accessed February 20, 2025). Steps included bias correction, segmentation into GM, WM, and CSF, normalization to MNI space via DARTEL (1 mm resolution), and smoothing with an 8 mm Gaussian kernel. Image quality was checked visually and using CAT12’s “Check Homogeneity” tool. Total intracranial volume (TIV) was computed to control for individual brain size differences. VBM used GM and WM density maps for tissue volume estimates. CT was measured with CAT12 and the Desikan-Killiany-Tourville (DKT) atlas (Farokhian et al. 2017, Klein and Tourville 2012). We also calculated Normalized GM, Normalized WM, and Normalized CSF for each sample by dividing the volumes of GM, WM, and CSF by the TIV and multiplying by 100.

### Brain age estimation framework

To quantify brain aging in EC and LC participants, we applied a pre-trained brain age prediction model developed from three large datasets: IXI (*N* = 563), OASIS (*N* = 313), and PPMI (*N* = 198) (Beheshti et al. 2024). A total of 1,054 healthy T1-weighted MRI scans were used, split into a training set (90%, *N* = 949) and a validation set (10%, *N* = 105). The model used support vector regression with a linear kernel, incorporating structural brain measures—GM, WM, and CSF volumes—along with scanner type, magnetic field strength, TIV, and sex as covariates. Chronological age was the target variable. Bias correction followed a validated procedure (Beheshti et al. 2019).

Briefly, the proposed bias-adjustment method employs a linear regression model to correct age-related bias in brain age predictions. The model explores the relationship between brain-PAD (Predicted Age – Chronological Age) and chronological age using the training set. An offset is computed for each sample using the following equation:

$$\:\mathrm{Offset}=\alpha\:\cdot\:{\Omega\:}+\beta\:$$

where $\:\alpha\:$is the slope and $\:\beta\:$is the intercept from the regression model. This offset, based on the individual’s actual age $\:{\Omega\:}$, is then subtracted from the predicted brain age to yield a bias-free estimate, validated across independent test sets. A sample code of this technique, along with comparisons to state-of-the-art methods, is available at: https://github.com/Beheshtiiman2/Bias-Correction-in-Brain-Age-Estimation-Frameworks. Model performance was strong, with a mean absolute error (MAE)—the average difference between predicted and actual age—of 4.72 years, and root mean squared error (RMSE)—the square root of the average squared differences—of 6.07 years in the training set. In the validation set, MAE was 4.63 years and RMSE was 5.88 years (Beheshti et al. 2019). Brain age was summarized using Brain-PAD (Mishra et al. 2021), where positive values indicate accelerated aging and negative values indicate preserved brain health (Sone and Beheshti 2022).

### Statistical analysis

Group comparability was assessed using independent-samples t-tests for continuous variables and χ² tests for categorical variables. Effect sizes were reported as Cohen’s d (t-tests) and Cramer’s V (χ²). Homogeneity of variance was evaluated using Levene’s test where applicable. For regression models, residual diagnostics were inspected to verify homoscedasticity, ensuring that variance was comparable across predictor values. All tests were two-tailed with α = 0.05.

#### **Sample size considerations**

This study is a secondary analysis of a fixed, publicly available dataset; therefore, an a priori sample-size calculation was not feasible. We therefore report a sensitivity analysis for the matched two-group design. With *n* = 68 per group (total *N* = 136), α = 0.05 (two-tailed), and 80% power, the minimum detectable standardized mean difference is Cohen’s *d* = 0.48. Given the additional loss of power associated with multiple-comparison correction in whole-brain analyses, the study may be underpowered to detect small effects, underscoring the need for replication in larger samples.

#### VBM analyses

Whole-brain analyses of GM and WM volumes were conducted using SPM25 (https://www.fil.ion.ucl.ac.uk/spm/software/spm25/, accessed February 20, 2025). Independent two-sample t-tests compared EC and LC groups. Confirmatory analyses applied voxel-wise family-wise error (FWE) correction at *p* < 0.05 to control Type I error across the whole brain. No clusters survived this threshold. Pre-specified exploratory analyses were then conducted using a voxel-level threshold of *p* < 0.001, with cluster-level false discovery rate (FDR) correction at *q* < 0.05, balancing sensitivity and specificity. Age, sex, and TIV were included as covariates.

Voxel-wise multiple regression analyses examined associations between GM/WM volumes and behavioral measures, including ChQ-ME, ChQ-AM, ESS, and PSQI. Age, sex, and TIV were included as covariates. Confirmatory FWE-corrected analyses were supplemented by exploratory analyses using the same voxel-level *p* < 0.001 and cluster-level FDR *q* < 0.05 thresholds. Standardized regression coefficients were reported to quantify effect sizes.

#### Cortical thickness analyses

Linear regression models compared EC and LC groups:

$$\:{\mathrm{CT}}_{\mathrm{region}}\sim\:{\beta\:}_{0}+{\beta\:}_{1}\cdot\:\mathrm{Cohort}+{\beta\:}_{2}\cdot\:\mathrm{Age}+{\beta\:}_{3}\cdot\:\mathrm{Sex}+\epsilon$$

where CT_region is the dependent variable, Cohort differentiates EC vs. LC, and ε represents residual error. Age and sex were included as covariates. FDR correction was applied across all regions to account for multiple comparisons. In addition, regression analyses were applied to CT measures to examine associations with behavioral and sleep-related variables (ChQ-ME, ChQ-AM, PSQI, ESS). Age and sex were included as covariates, and FDR correction was applied across all regions. Significant associations were summarized, highlighting cortical regions where thickness correlated with chronotype and sleep measures. Consistent with established recommendations (Goto et al. 2022), TIV was not included as a covariate in the CT analyses.

**Fig. 1:**
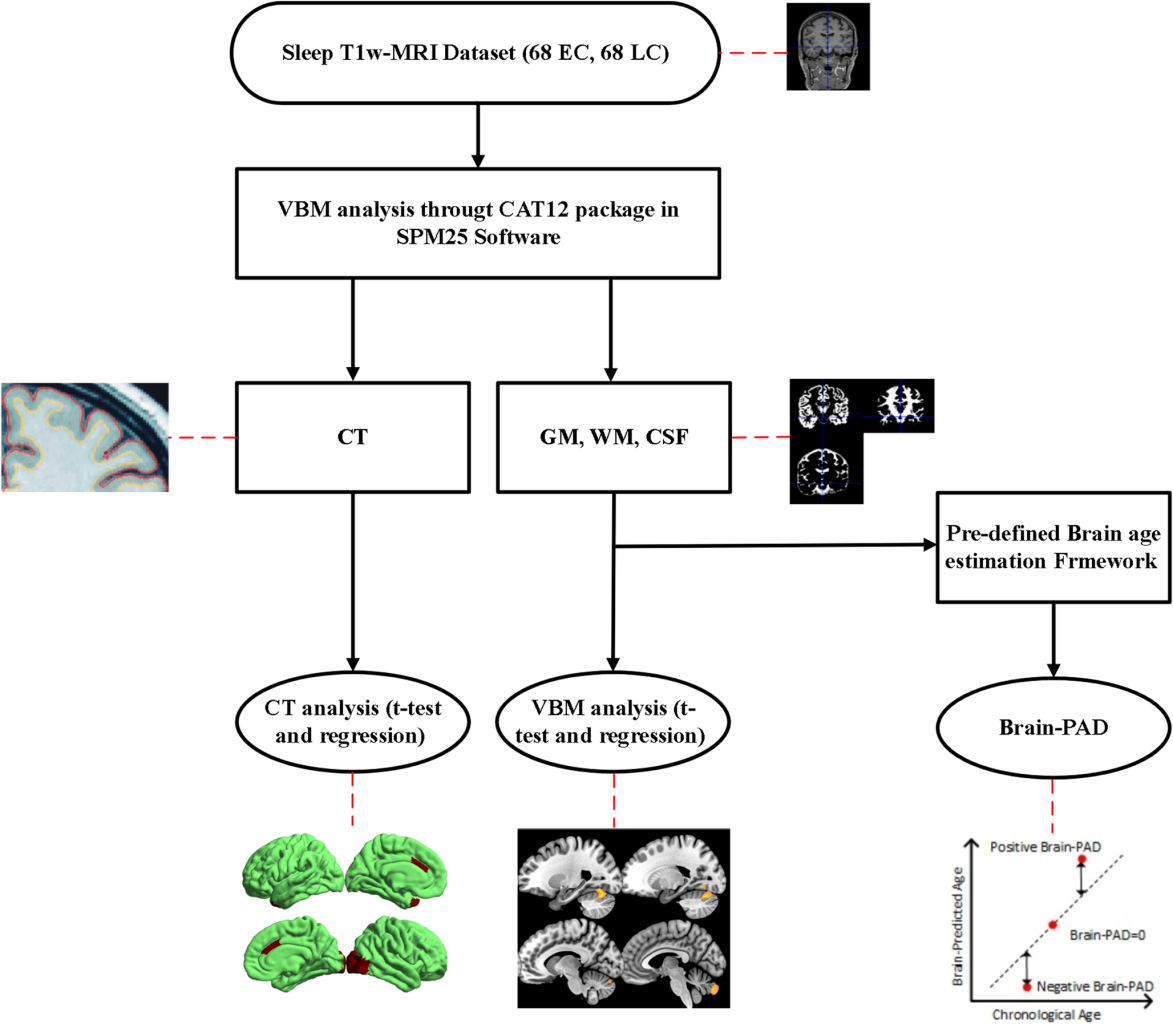
Workflow of Brain Structural Analysis and Brain Age Estimation in Sleep Cohort. The figure illustrates the processing pipeline used in the study. The dataset consisted of 68 Early Chronotype (EC) and 68 Late Chronotype (LC) participants. Structural MRI scans were analyzed using the VBM pipeline through the CAT12 toolbox in SPM25. Cortical thickness (CT) and tissue volumes including gray matter (GM), white matter (WM), and cerebrospinal fluid (CSF) were extracted. CT measures were analyzed using t-tests and regression, and GM/WM/CSF volumes were similarly analyzed. In parallel, a pre-defined brain age estimation framework was applied to compute Brain-PAD (Predicted Age Difference), which quantifies deviations between predicted brain age and chronological age

Figure 1 illustrates the T1-weighted MRI preprocessing pipeline and the statistical analyses applied to our dataset.

## Results

Across primary whole-brain voxel-wise analyses, no between-group structural differences survived family-wise error (FWE) correction for multiple comparisons (*p* < 0.05). Therefore, we report any patterns detected only in the pre-specified exploratory framework (voxel-wise *p* < 0.001, uncorrected; cluster-level FDR q < 0.05) as exploratory findings requiring independent replication, and we interpret them cautiously without implying robust group effects.

### Clinical and demographic characteristics

Participant characteristics are summarized in Table 1. The EC and LC groups were well matched on age and sex and did not differ on ChQ-AM, PSQI, or ESS (all *p* > 0.05). As expected given the group definition, ChQ-ME scores differed markedly between groups (*p* < 0.0001), confirming strong separation in diurnal preference and validating the EC/LC classification used in subsequent analyses. These results support the group classification and reduce the likelihood that subsequent findings are driven by demographic differences or general sleep quality/sleepiness rather than chronotype.

**Table 1:** Demographic and clinical information for 68 EC and 68 age- and sex-matched LC participants

Variable	EC (*N* = 68)	LC (*N* = 68)	t / χ²	*p*-value	Effect size
Male, n (%)	23 (33.8%)	26 (38.2%)	χ² = 0.13	0.72	V = 0.031
Age, years	24.80 ± 3.90	23.85 ± 3.51	t = 1.50	0.12	d = 0.256
ChQ-ME	16.76 ± 2.69	26.67 ± 2.61	t = -21.78	< 0.0001	d = -3.739
ChQ-AM	20.42 ± 3.97	21.19 ± 3.49	t = -1.19	0.23	d = -0.206
PSQI	2.94 ± 1.26	3.21 ± 1.52	t = -1.10	0.27	d = -0.193
ESS	6.79 ± 3.16	7.33 ± 3.83	t = -0.90	0.36	d = -0.154

*EC *early chronotype, *LC* late chronotype, *ChQ-ME* morningness–eveningness scale of the Chronotype Questionnaire, *ChQ-AM *amplitude scale of the Chronotype Questionnaire, *PSQI* Pittsburgh Sleep Quality Index, *ESS* Epworth Sleepiness Scale. Continuous variables were compared using independent-samples t-tests (Welch-corrected when variances were unequal), and categorical variables were compared using χ² tests. Effect sizes are reported as Cohen’s d (t-tests; computed as EC–LC) and Cramer’s V (χ²). No significant differences were observed for age, sex, ChQ-AM, PSQI, or ESS. As expected based on the group definition, ChQ-ME differed markedly between EC and LC participants, confirming clear chronotype separation

### VBM analysis

**Fig. 2:**
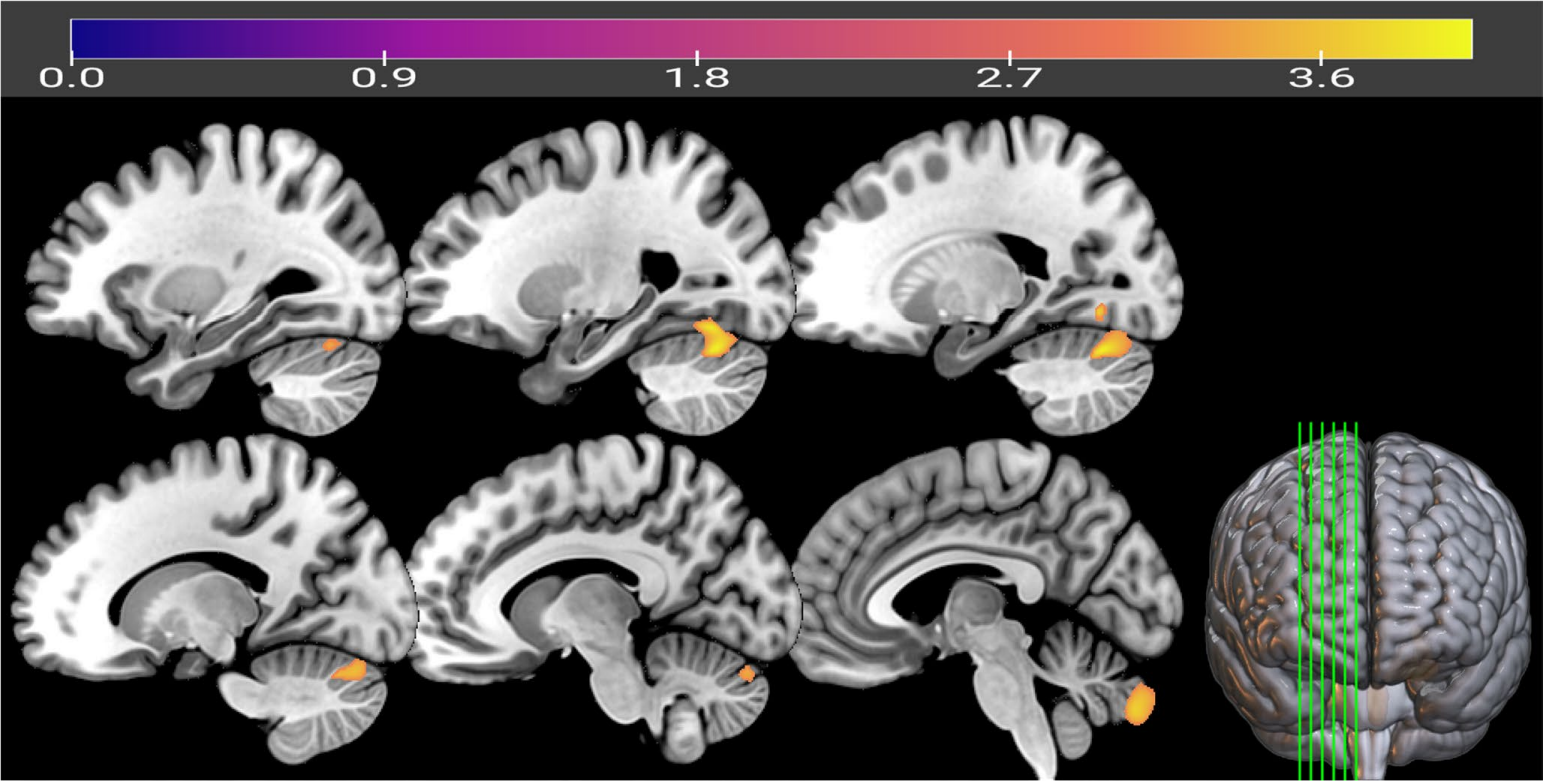
Exploratory gray matter cluster showing lower volume in late chronotype (LC) relative to age- and sex-matched early chronotype (EC) participants, identified using voxel-based morphometry (VBM) in SPM25. Whole-brain voxel-wise analyses did not yield effects that survived FWE correction (*p* < 0.05). Under the pre-specified exploratory framework (voxel-wise *p* < 0.001, uncorrected; cluster-level FDR q < 0.05), one cluster was detected, centered in the left cerebellum (posterior lobe/declive) and extending into adjacent occipital/fusiform regions (see Table 2 for cluster statistics). For visualization, the T-map is displayed at voxel-wise *p* < 0.0001 and cluster extent > 500 voxels (uncorrected). Warm colors indicate higher T values for the LC < EC contrast; the color bar denotes T value

**Fig. 3:**
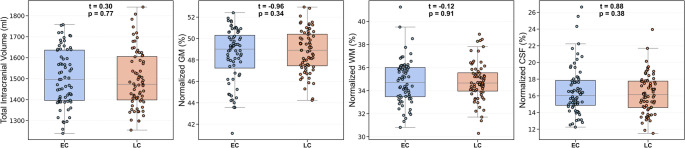
Boxplots of total intracranial volume (TIV), normalized gray matter (GM), normalized white matter (WM), and normalized cerebrospinal fluid (CSF) across early chronotype (EC) and late chronotype (LC) groups. Group comparisons were performed using independent-samples t-tests (two-tailed), with t-statistics and p-values annotated for each measure. No between-group differences were detected for any measure (all *p* ≥ 0.34)

Primary whole-brain voxel-wise analyses did not identify significant between-group differences in gray or white matter volumes that survived FWE correction (*p* < 0.05). As pre-specified in the Methods, we then examined patterns under an exploratory inference framework (voxel-wise *p* < 0.001, uncorrected; cluster-level FDR q < 0.05). Under this exploratory framework, LC participants showed one cluster with lower GM volume relative to EC participants, centered in the left cerebellum (posterior lobe/declive) and extending into crus I and adjacent occipital/fusiform regions (cluster size = 4,097 voxels; cluster-level FDR q = 0.005; peak T = 4.05; MNI: − 22, − 69, − 24; Table 2; Fig. 2). No clusters indicating WM volume differences were identified under the exploratory framework. Voxel-wise multiple regression analyses testing associations between GM/WM volumes and behavioral measures (ChQ-ME, ChQ-AM, PSQI, ESS) did not yield significant results after correction for multiple comparisons (FWE *p* < 0.05) or cluster-level FDR (q < 0.05). Additionally, the EC and LC groups did not differ in TIV, normalized GM, normalized WM, or normalized CSF (all *p* > 0.38; Fig. 3).

**Table 2:** Exploratory gray matter cluster showing lower volume in LC relative to EC (VBM; voxel-wise *p* < 0.001 uncorrected; cluster-level FDR q < 0.05)

Region	Cluster Size (No. of Voxels)	q (FDR)	Hemisphere	MNI Coordinates (x, y, z)	T Value (Peak Voxel)
Left cerebellum (posterior lobe/declive/crus I), extending into occipital and fusiform regions	4097	0.005	Left	-22, -69, -24	4.05

*MNI* Montreal Neurological Institute, *FDR* false discovery rate. The peak voxel is the voxel with the maximum T value within the cluster. The cluster was identified under the pre-specified exploratory framework (voxel-wise *p* < 0.001, uncorrected; cluster-level FDR q < 0.05). No clusters indicating WM volume differences were detected between groups under the same framework

### Cortical thickness analysis

**Fig. 4:**
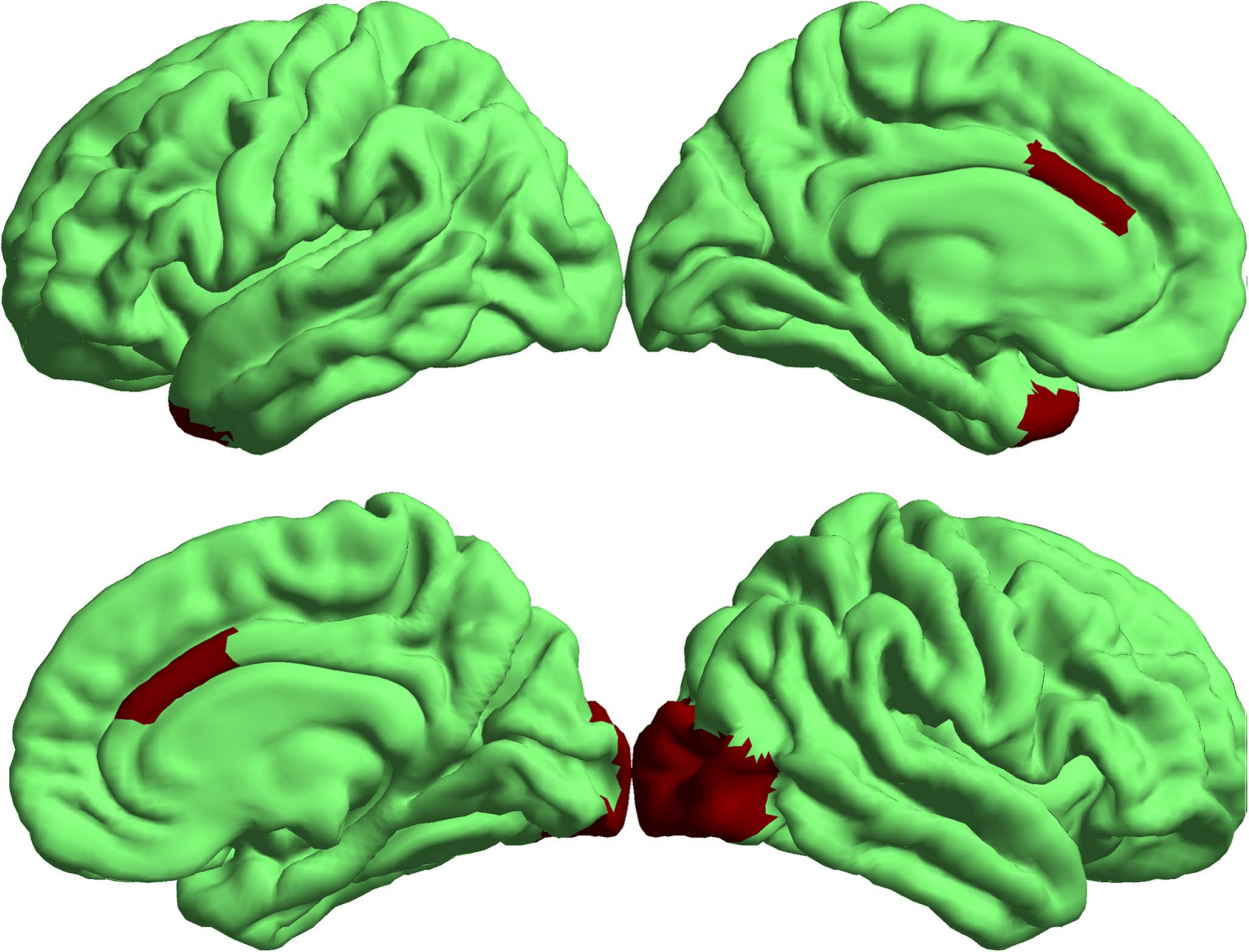
Nominal cortical thickness (CT) differences between early (EC) and late chronotype (LC) participants, displayed on inflated cortical surfaces. Group effects were estimated using region-wise regression models adjusting for age and sex (see Table 3). At a nominal level, LC participants showed thinner CT in the bilateral caudal anterior cingulate cortex and the right lateral occipital cortex, whereas EC participants showed thinner CT in the left temporal pole. None of these regional effects survived FDR correction across the four regions (all q > 0.15)

**Fig. 5:**
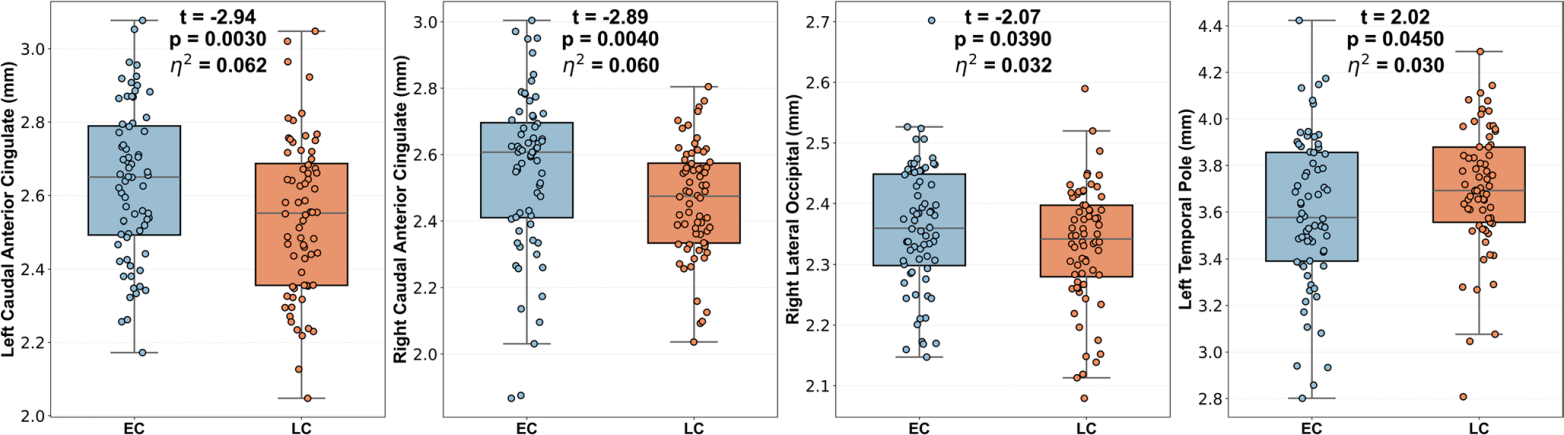
Boxplots illustrating nominal cortical thickness differences between early chronotype (EC) and late chronotype (LC) participants across regions identified in the region-wise regression models (adjusted for age and sex; *n* = 136; see Table 3). Statistical values (t, p, and partial η²) are shown within each panel. These effects are nominal and did not survive FDR correction across the four regions (all q > 0.15)

Region-wise group comparisons (adjusting for age and sex) identified nominal CT differences between EC and LC participants in a small set of regions (Table 3). Figure 4 shows the nominal cortical thickness differences between EC and LC participants on inflated surfaces, while Fig. 5 provides boxplots illustrating these differences across the identified regions. Relative to EC, the LC group showed nominally thinner CT in the bilateral caudal ACC and the right lateral occipital cortex, whereas EC showed nominally thinner CT in the left temporal pole. Effect sizes (partial η²) were small (range: 0.03–0.06). However, none of these regional effects survived FDR correction across regions (all q > 0.05), and they are therefore interpreted as exploratory. Age was a significant covariate in three of the four regions (β range: − 0.012 to − 0.007, *p* < 0.02), consistent with expected age-related cortical thinning; sex effects were limited to the right caudal ACC (β = 0.074, *p* = 0.041). Of note, mean cortical thickness did not differ between the EC (2.63 ± 0.07 mm) and LC (2.64 ± 0.05 mm) groups, with no evidence of a meaningful group difference (*p* ≈ 1).

**Fig. 6:**
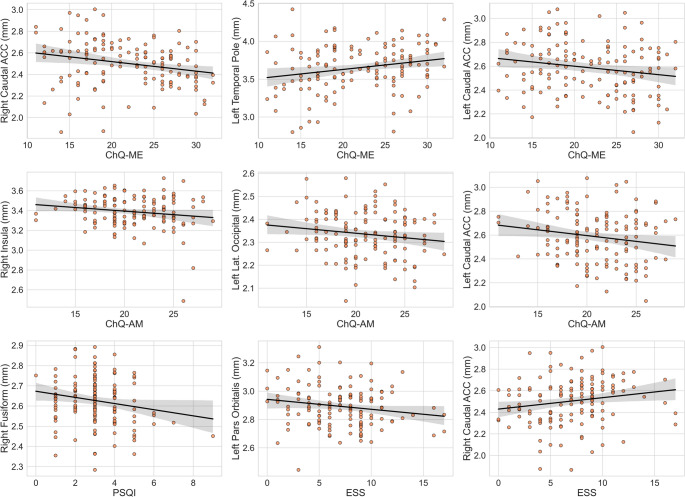
Scatter plots (with fitted regression lines) illustrating nominal associations between cortical thickness and questionnaire measures of chronotype and sleep (ChQ-ME, ChQ-AM, PSQI, ESS). Each panel corresponds to a region-wise linear regression model in which cortical thickness in the indicated region was related to the questionnaire score, adjusting for age and sex. Panels are shown for models with nominal *p* < 0.05 across the region-wise analyses (68 regions). None of the associations survived FDR correction for multiple comparisons (all q > 0.07); therefore, these patterns should be interpreted as exploratory

To complement group comparisons, we conducted exploratory region-wise regression analyses examining associations between CT and questionnaire measures (ChQ-ME, ChQ-AM, PSQI, ESS). At a nominal level, higher ChQ-ME scores (greater eveningness) were associated with thinner CT in the bilateral caudal ACC and right lateral occipital cortex, whereas CT in the left temporal pole showed a positive association with ChQ-ME in the opposite direction (Fig. 6; effect estimates and confidence intervals are reported in Table 4). Additional nominal associations were observed between CT and ChQ-AM, PSQI, and ESS across several regions. None of these associations survived FDR correction for multiple comparisons (all q > 0.05), and they are therefore reported as exploratory patterns requiring independent replication.

**Table 3:** Region-wise regression models predicting cortical thickness from chronotype group, age, and sex. (nominal p-values)

Predictor	Left Caudal ACC	Right Caudal ACC	Right Lateral Occipital	Left Temporal Pole
Group (LC vs. EC)	β = − 0.106, t = − 2.95, *p* = 0.0037	β = − 0.100, t = − 2.89, *p* = 0.0044	β = − 0.035, t = − 2.08, *p* = 0.039	β = 0.105, t = 2.02, *p* = 0.045
Age	β = − 0.012, t = − 2.51, *p* = 0.013	β = − 0.013, t = − 2.85, *p* = 0.005	β = − 0.007, t = − 3.19, *p* = 0.001	β = 0.004, t = 0.61, *p* = 0.544
Sex	β = − 0.019, t = − 0.52, *p* = 0.602	β = 0.073, t = 2.06, *p* = 0.041	β = − 0.002, t = − 0.16, *p* = 0.871	β = 0.013, t = 0.25, *p* = 0.801

*ACC* anterior cingulate cortex. Outcome: cortical thickness. Regression models included age and sex as covariates (sex coded 0 = female, 1 = male). β denotes standardized regression coefficients. After FDR correction across the four regional group effects, none of the group coefficients remained significant (all q > 0.15)

**Table 4:** Exploratory region-wise regression models relating questionnaire measures cortical thickness (adjusted for age and sex)

**Outcome**	**Brain Region**	**β** **(CT)**	**t**	**P**	**β** ** (Age)**	**t**	**p**	**β** ** (Sex)**	**t**	**p**
ChQ-ME	Right Caudal Anterior Cingulate Cortex	-7.584	-3.321	0.001	-0.286	-2.235	0.027	0.858	0.871	0.386
ChQ-ME	Left Temporal Pole	4.172	2.671	0.009	-0.208	-1.641	0.103	0.225	0.229	0.819
ChQ-ME	Left Caudal Anterior Cingulate Cortex	-5.620	-2.516	0.013	-0.256	-1.982	0.050	0.180	0.182	0.856
ChQ-ME	Right Lateral Occipital Cortex	-9.782	-2.034	0.044	-0.264	-1.995	0.048	0.249	0.251	0.802
ChQ-AM	Right Insula	-4.811	-2.380	0.019	-0.128	-1.470	0.144	1.285	1.969	0.051
ChQ-AM	Left Lateral Occipital Cortex	-7.296	-2.235	0.027	-0.102	-1.200	0.232	1.448	2.192	0.030
ChQ-AM	Left Caudal Anterior Cingulate Cortex	-3.161	-2.129	0.035	-0.110	-1.275	0.205	1.185	1.810	0.073
PSQI	Right Fusiform gyrus	-2.198	-2.096	0.038	-0.024	-0.729	0.467	0.198	0.789	0.432
ESS	Left Pars Orbitalis	-4.702	-2.195	0.030	-0.072	-0.887	0.377	0.820	1.324	0.188

*ChQ-ME* morningness–eveningness scale of the Chronotype Questionnaire, *ChQ-AM* amplitude scale of the Chronotype Questionnaire, *PSQI* Pittsburgh Sleep Quality Index, *ESS* Epworth Sleepiness Scale. Each row reports a separate regression model with the questionnaire measure as the outcome and cortical thickness in the specified region as the predictor, adjusting for age and sex. Coefficients are unstandardized (B). Rows are shown for nominal associations (p < 0.05). None of the associations survived false discovery rate (FDR) correction for multiple comparisons (all q > 0.07)

### Brain age estimation

Brain age predicted from MRI data showed a positive correlation with chronological age (Pearson’s *r* = 0.60, *p* < 1 × 10⁻⁵), supporting expected age–prediction alignment in this sample. No between-group difference was detected in Brain-PAD between the LC (M = 0.6 ± 4.3 years) and EC (M = 0.4 ± 5.2 years) groups (t(134) = − 1.45, *p* = 0.15). Thus, in this cohort of healthy young adults, Brain-PAD did not differ by chronotype at the group level.

To assess whether Brain-PAD was associated with chronotype preference, we fitted a multiple linear regression with ChQ-ME as the outcome and Brain-PAD as the predictor of interest, adjusting for age and sex (ChQ-ME ~ Brain-PAD + age + sex). The overall model was not significant (R² = 0.03; F(3,132) = 1.18, *p* = 0.32), and Brain-PAD was not associated with ChQ-ME (β = 0.11, 95% CI [–0.10, 0.31], *p* = 0.29). Age and sex were also non-significant covariates. These results suggest that Brain-PAD was not related to chronotype preference in this sample, despite the exploratory regional patterns observed in GM volume and cortical thickness.

## Discussion

It is well documented that chronotype is associated with distinct behavioral, physiological, and genetic characteristics, as well as differences in functional brain activity (Jones et al. 2019, Salehinejad et al. 2021, Kossowski et al. 2021, Chauhan et al. 2025). However, the relationship between chronotype and brain structure remains incompletely characterized, particularly in young adults. Prior structural MRI studies have reported chronotype-related differences across distributed cortical and subcortical regions, yet findings remain heterogeneous in measures, analytic strategies, and correction approaches and are often based on a single morphometric modality (Horne and Norbury 2018, Horne and Norbury 2018, Evans et al. 2021). Here, we used a multimodal structural framework—combining VBM, CT, and brain-predicted age/Brain-PAD—to provide an integrated assessment within the same healthy young-adult cohort and to test our a priori hypotheses using both matched extreme-group comparisons and continuous chronotype sensitivity analyses. While the most conservative multiple-comparison corrections attenuated statistical significance, convergent spatial patterns across modalities pointed to subtle, region-specific variation associated with late chronotype, offering mechanistically interpretable targets for future preregistered and longitudinal work.

Our first hypothesis was that LC individuals would exhibit regionally specific reductions in GM volume and cortical thinning, particularly in the ACC, occipital cortex, and posterior cerebellar lobules. This hypothesis was partially supported. The VBM analysis revealed reduced GM volume in LC participants relative to EC within the left posterior cerebellum, encompassing the declive, posterior lobe, and Crus I, extending into the fusiform and occipital cortices (Table 2; Fig. 2). CT analysis, while not surviving FDR correction (q > 0.15), showed nominally thinner cortex in the bilateral caudal ACC and right lateral occipital cortex in LC participants, whereas EC individuals exhibited thinner cortex in the left temporal pole (Table 3).

The regional convergence across the posterior cerebellum, ACC, and occipital cortices supports the anatomical specificity of chronotype-related variation (Salehinejad et al. 2021, Horne and Norbury 2018). These findings converge partially with previous reports identifying posterior cortical and cerebellar involvement in chronotype differences (Horne and Norbury 2018, Evans et al. 2021), but differ from studies emphasizing thalamic or frontal volume effects (Salehinejad et al. 2021). Our exploratory pattern involving occipital/cerebellar regions is broadly consistent with prior reports linking eveningness/chronotype to regional occipital gray matter differences (e.g., Evans et al. 2021) and to cortical structural variation across sleep timing preference (Rosenberg et al. 2018), while heterogeneity across studies likely reflects differences in sample characteristics, chronotype operationalization, and correction strategies.

The posterior cerebellum—particularly Crus I—is increasingly recognized for its role in higher-order cognitive functions, including working memory, executive control, and affect regulation (Botvinick et al. 2004, Bush et al. 2000). The structural vulnerability of the ACC among LC individuals is consistent with evidence suggesting reduced fronto-limbic connectivity in evening types, and may reflect diminished regulatory capacity even in the absence of clinical symptomatology (Botvinick et al. 2004, Bush et al. 2000). The finding of right lateral occipital thinning adds to emerging evidence that posterior cortical regions may be sensitive to circadian influences, potentially due to their involvement in visual processing and responsiveness to light cues that modulate circadian entrainment and melatonin suppression (Grill-Spector et al. 2001, Malach et al. 1995, Lucas et al. 2014). Reduced GM and CT in LC individuals may thus reflect subtle neuroplastic adaptations or vulnerabilities associated with chronic circadian misalignment. Evening chronotypes are more likely to experience irregular light exposure, delayed sleep timing, and social jet lag, leading to persistent misalignment between internal circadian rhythms and environmental schedules (Gentry 2021, Taillard et al. 2021). Such repeated desynchronization could induce small but measurable effects on synaptic density or glial structure, particularly in high-metabolic-demand regions such as the ACC and cerebellum. The nominal thinning in the right lateral occipital cortex further suggests sensitivity of posterior visual regions to circadian influences, potentially linked to differential light exposure patterns that affect melatonin suppression and photic entrainment (Grill-Spector et al. 2001, Malach et al. 1995, Lucas et al. 2014). Interestingly, EC participants displayed thinner cortex in the left temporal pole, a region implicated in social–emotional integration. This bidirectional pattern implies that chronotype-related neural variation may not be strictly disadvantageous but rather reflects distinct behavioral and environmental adaptations. Our findings contrast with other studies that reported reduced GM in posterior cortical regions among early chronotypes (Rosenberg et al. 2018). This discrepancy may stem from methodological differences, including the use of categorical vs. continuous chronotype classification, participant demographics (e.g., sex distribution), and neuroimaging pipelines. More specifically, studies using different chronotype instruments (e.g., MCTQ-based mid-sleep metrics versus questionnaire-based preference) and different correction thresholds have variably emphasized thalamic/frontal versus posterior cortical and cerebellar effects, suggesting that operationalization and analytic stringency may shape the apparent regional pattern (Horne and Norbury 2018, Horne and Norbury 2018, Evans et al. 2021, Gentry 2021). Nonetheless, both studies highlight the occipital cortex as a chronotype-sensitive region, reinforcing its relevance in the study of circadian neurobiology (Evans et al. 2021, Rosenberg et al. 2018). Collectively, these findings provide partial support for our first hypothesis, indicating that later chronotype is associated with subtle, spatially coherent structural differences within networks subserving emotional regulation and circadian processing.

Next, we hypothesized that LC individuals would show greater deviation between predicted and chronological brain age, reflecting accelerated structural aging or delayed neurodevelopment. This hypothesis was not supported. The mean Brain-PAD did not differ significantly between chronotypes (LC: 0.6 ± 4.3 years; EC: 0.4 ± 5.2 years; *p* = 0.15). These findings indicate that, at a global level, both chronotype groups exhibit comparable structural aging trajectories in early adulthood.

The absence of a chronotype-related Brain-PAD effect suggests that neural differences associated with eveningness are regionally confined rather than widespread markers of accelerated aging. It is plausible that chronotype-related divergence in biological brain age may emerge later in life, when cumulative circadian disruption or lifestyle-related stressors begin to exert broader neural consequences. In young adults—who typically maintain structural and metabolic resilience—the observed cerebellar and cortical thinning may represent early-stage or compensatory remodeling rather than degenerative change. Methodologically, this null result also reflects the complementary sensitivity of imaging metrics. Brain age models capture diffuse, macrostructural aging signatures, whereas VBM and CT detect focal regional variation. Our multimodal approach therefore demonstrates that chronotype exerts localized structural effects not yet manifest in whole-brain aging indices. The absence of group differences in WM volume further supports this interpretation, as WM alterations typically accompany later or more global aging processes. Taken together, these findings do not support accelerated aging among late chronotypes in young adulthood. Instead, they point to regionally specific morphological adaptations within neural systems sensitive to circadian behavior, independent of generalized structural decline.

Our last hypothesis was that sleep-related measures would correlate with structural differences, particularly within regions showing chronotype-dependent effects. This hypothesis was not statistically confirmed, though exploratory analyses revealed several plausible trends. Voxel-wise regressions revealed no significant associations between GM or WM volume and behavioral indices (ChQ-ME, ChQ-AM, PSQI, ESS) after FDR correction. CT analyses, however, identified nominal associations between thinner caudal ACC and right lateral occipital cortex and lower morningness scores, as well as between reduced circadian amplitude or poorer subjective sleep quality and thinner cortex in the insula, fusiform, and pars orbitalis (Table 3). None of these associations survived FDR correction and should therefore be interpreted cautiously. Several considerations help contextualize these results. First, VBM and CT reflect distinct neurobiological processes: VBM indexes local tissue concentration, whereas CT quantifies laminar integrity and synaptic density. It is conceivable that subtle laminar changes precede detectable volumetric variation, making CT marginally more sensitive to early cortical remodeling in young adults. Second, both techniques involve a high multiple-comparison burden, which greatly reduces statistical power to detect small but consistent effects. Third, self-reported measures such as PSQI and ESS capture subjective experience rather than objective circadian physiology and thus fail to detect the degree of misalignment driving neural changes. Biologically, the direction of the nominal effects—thinner ACC and occipital cortices associated with eveningness—is consistent with prior work linking these regions to emotional regulation and photic entrainment (Zhou et al. 2024, García-Cabezas and Barbas 2017, Perlman and Pelphrey 2010). The fact that these patterns only emerged at uncorrected thresholds suggests that chronotype-related neural variation in young adults is subtle, potentially reflecting stable trait characteristics rather than transient consequences of sleep deprivation. Future research should incorporate objective measures of circadian phase (e.g., melatonin onset, actigraphy, or light exposure metrics) alongside multimodal imaging to clarify causality. Longitudinal studies and larger samples will be essential to determine whether these small, regionally confined differences predict behavioral or cognitive outcomes over time. Moreover, pre-registered, region-of-interest analyses targeting the ACC, posterior cerebellum, and occipital cortex—guided by our current results—may offer a more sensitive test of these hypotheses than broad discovery analyses.

Although none of the reported effects survived stringent correction, the anatomical convergence across modalities suggests that chronotype is subtly embedded within the architecture of fronto-cerebellar and posterior cortical systems. These regions collectively support emotion regulation, cognitive control, and the integration of sensory and circadian information. The observed morphometric trends may thus represent early, trait-like signatures of individual differences in circadian preference, reflecting either developmental specialization or low-level adaptation to habitual sleep–wake timing. Importantly, these structural features were independent of self-reported sleep quality or daytime sleepiness, suggesting that chronotype’s neuroanatomical correlates are not merely consequences of poor sleep but may instead index intrinsic differences in circadian regulation and neural efficiency. From a translational perspective, these findings underscore that chronotype—often regarded as a behavioral preference—has measurable, neurobiological correlates even in healthy young adults. Understanding how such subtle differences evolve could inform preventive strategies aimed at minimizing the long-term effects of circadian misalignment on mental and cognitive health.

Several limitations should be acknowledged. First, the cross-sectional design precludes causal inference. Second, the relatively homogeneous young adult sample limits the generalizability of the findings to older or clinical populations, in which circadian disruption may exert more pronounced neural effects. Third, chronotype and sleep variables were derived from self-report measures, which may underestimate true circadian variability. Moreover, lifestyle factors such as caffeine consumption, light exposure, and physical activity were not controlled and could contribute to structural variability. The relatively modest sample size (*N* = 136; 68 per group) represents a major limitation given the high dimensionality of voxel-wise neuroimaging data. Even moderate effect sizes may remain undetected after multiple-comparison correction, limiting both sensitivity and generalizability. Larger, multi-site datasets are therefore essential to validate the subtle trends observed here and to determine their reproducibility across populations and analytical frameworks. Finally, although WM volume was included in the analyses to provide a broader perspective on chronotype-related brain structure, no significant group differences were observed. This null finding should be interpreted cautiously, as VBMA is relatively insensitive to WM architecture and may fail to capture microstructural alterations. Advanced imaging techniques such as diffusion tensor imaging (DTI), which provide more detailed assessments of WM integrity and connectivity, may be better suited to detect chronotype-related differences and should be prioritized in future research.

Together, these findings highlight that chronotype may represent an intrinsic neurobiological dimension influencing regional brain structure, potentially serving as an early marker of vulnerability to circadian misalignment.

## Conclusion

Our results reveal regionally specific cerebellar and cortical patterns consistent with chronotype-related neural variation, even though these effects did not survive stringent correction. Late chronotype was characterized by reduced cerebellar GM and thinner ACC and occipital cortices—regions integral to emotional regulation, executive control, and circadian alignment—while no global differences in Brain-PAD were detected. These findings highlight that chronotype may leave a subtle but measurable imprint on brain structure, emphasizing the importance of considering sleep–wake timing as a dimension of neural and cognitive health in young adulthood.

## Data Availability

This study is a secondary analysis of anonymized, publicly available data obtained from the OpenNeuro repository (https://openneuro.org/datasets/ds003826/versions/3.0.1/file-display/dataset_description.json; accessed March 1, 2025). No new data were collected by the authors. The original data were collected as part of two separate studies, both of which received ethical approval from the Research Ethics Committee at the Institute of Applied Psychology, Jagiellonian University, and the Bioethics Commission at the Polish Military Institute of Aviation Medicine. All participants gave written informed consent prior to participation in the original studies. Data collection complied with the ethical standards of the respective institutions and was conducted in accordance with the Declaration of Helsinki.
